# Forensic Genetic Analyses of Melanistic Iguanas Highlight the Need to Monitor the Iguanid Trade

**DOI:** 10.3390/ani12192660

**Published:** 2022-10-03

**Authors:** Blaklie Mitchell, Mark E. Welch, Matthijs P. van den Burg

**Affiliations:** 1 Department of Biological Sciences, Mississippi State University, Starkville, MS 39762, USA; 2 IUCN SSC Iguana Specialist Group, 1196 Gland, Switzerland; 3 Department of Biogeography and Global Change, Museo Nacional de Ciencias Naturales, Consejo Superior de Investigaciones Científicas, C/José Gutiérrez Abascal 2, 28006 Madrid, Spain; 4 BioCoRe S. Coop., Calle Villagarcía 6, 28010 Madrid, Spain

**Keywords:** CITES, common green iguana, fraudulent, *Iguana iguana*, *Iguana melanoderma*, international trade, pet trade, reptiles, wildlife trade

## Abstract

**Simple Summary:**

The illegal pet trade remains an ongoing, substantial threat to wild populations, especially small insular populations, and can even lead to extinction. Fraudulent activity within the global reptile trade is known to occur, but its identification through forensic applications depends on knowledge of diversity within wild populations. In this study, we assessed the geographic origin of melanistic iguanas (Iguana iguana), which are only found in nations that have never authorized legal export of live animals. Analysis of genetic data from two pet iguanas in the USA flag these as originating from Saba or Montserrat, from which no export permits have ever been issued, confirming their illegal origin. Despite the international trade in I. iguana, in which tens- if not hundreds of thousands of specimens are traded each year, only a handful of individuals have been genetically assessed. Our work highlights the utility of applying forensic genetic techniques to this trade in order to track and discourage illegal activity.

**Abstract:**

Lizards within the *Iguana iguana* species complex are among the most common reptilian pets, with the widest natural geographic range among iguanids. Deep phylogenetic divergence distinguishes multiple mitochondrial clades, and several taxonomic changes have recently been proposed. These small populations, typically island endemics, are threatened by numerous factors, including the international pet trade. Recent investigations reveal the absence of required CITES permits for lawful export of animals, providing evidence of ongoing illegal trade. Additional monitoring of trade in iguanas can be achieved through the application of forensic molecular techniques. In this study, two captive melanistic iguanas were genotyped for molecular markers for which geographic distributions of alleles have been established. Mitochondrial sequencing indicates that both animals carry a haplotype known to originate from the islands of Saba and Montserrat, populations taxonomically proposed to be *Iguana melanoderma*. Genotypes at 15 microsatellite loci are equally consistent with this origin, given the results of a principal component analysis. This first forensic genetic assessment within the extensive *I. iguana* pet trade highlights the presence of illegal activity. The need for additional forensic assessments of pet-trade iguanas is evident, especially given that their value is driven by variety and rarity, which is further intensified by recent taxonomic changes.

## 1. Introduction

The international pet trade threatens taxa across the Tree of Life [1,2,3], including reptiles [4]. The trade in reptiles impacts wild populations and concerns both trade-regulated and unregulated species [4,5,6]. Forensic applications, including genetic analyses [7], have been utilized for numerous species to identify the geographic origin of confiscated or traded individuals in an effort to detect illegal activity [8] and, occasionally, to repatriate specimens [9,10]. Data gathered as a result of these efforts are crucial to understand trade dynamics and to provide insights for regulatory improvements.

The *Iguana iguana* species complex is the most widespread among iguanids [11]. Native populations within this complex persist throughout mainland Central and South America and on multiple Caribbean islands. Despite the widespread occurrence of the complex, only two species are widely recognized within the *Iguana* genus: *I. delicatissima* and *I. iguana* [11]. Currently, IUCN guidelines designate *I. delicatissima* as ‘critically endangered’, whereas *I. iguana* is of ‘least concern’ [12,13]. Recently, several insular *I. iguana* populations in the Caribbean Lesser Antilles were proposed for full species recognition: *I. insularis*, constituting native populations from St. Lucia, St. Vincent and the Grenadines and Grenada, as well as *I. melanoderma*, which includes native melanistic populations from Saba, Montserrat, and St. Croix and St. Thomas [14,15,16]. However, these populations are currently designated as subspecies, with only *I. iguana insularis* (St. Vincent and the Grenadines and Grenada) and *I. i. sanctaluciae* (St. Lucia) recognized by the Iguana Taxonomy Working Group, whereas *I. melanoderma* is still considered part of *I. i. iguana* (for details, see ITWG [17]). Although these remain currently unassessed under IUCN guidelines, the numerous threats faced by these island *I. iguana* populations is likely to result in them being designated as threatened [14,16,18].

Despite its ‘least concern’ assignment, regional assessments across *I. iguana* indicate that local populations can be threatened due to a range of factors. Whereas mainland populations are also locally endangered [19,20], the most at-risk are insular populations in the Lesser Antilles, given a range of prominent threats, e.g., hunting, invasive species, and the spread of non-native iguanas [12,14,15,21,22]. Overall, *I. iguana* are among the most traded reptiles globally [23,24], with native populations being harvested for trade, often without restrictions [20,25]. The lack of proper management practices and apparently unrestricted access to endemic populations of *I. iguana* represents a rapidly growing problem within the conservation community that must be addressed with accessible, quantitative data.

Since 1977, the trade in *Iguana* sp. has been regulated through the Convention on International Trade in Endangered Species of Wild Fauna and Flora (CITES), listed in Appendix II. Despite trade regulation, trafficking of *Iguana* sp. has been reported from the Central American mainland [20,26] and, recently, from Caribbean islands [27,28]. However, only three pet-trade iguanas have ever been included in genetic studies [29,30], and no study to date has applied forensic genetics to captive iguanas. The Lesser Antilles, in particular, have emerged as a trade hub for iguanas, presumably given the native and non-native diversity found there. Although all islands had native *Iguana* populations, historically, the majority has either been extirpated or replaced by non-native iguanas, with few native populations remaining [18]. As established for other species [31,32,33], iguanas can be fraudulently presented as captive bred to mask their wild or geographic origin. Traders appear to exploit this situation by illegally transporting iguanas from native populations to islands with non-native populations. In the case of latter situation, these introduced iguanas are generally not protected, given their alien origin and invasive character; therefore, restrictions on trading and captive breeding of iguanas seem to be nearly absent, and CITES permits can easily be acquired [27,28].

Recently, dealers have used such methods to remove and export melanistic iguanas (proposed as *Iguana melanoderma* [15]) out of the Lesser Antilles [27,28]. Although melanistic iguanas are globally advertised as originating from the island of Saba, which is corroborated by investigative studies [27,28], no study has yet identified their genetic and geographic origin. This study serves as a first forensic effort to begin characterizing the genetic signature of melanistic iguanas acquired through the pet trade to substantiate their putative origin.

## 2. Materials and Methods

We obtained two blood samples from a breeding pair of melanistic iguanas (proposed as *Iguana melanoderma* by Breuil et al. [15]) from a USA-based pet trader that are advertised, as well as their offspring for sale, as Saban Black Iguanas. DNA was isolated using Maxwell^®^ 16 Tissue DNA Purification kits following the manufacturer’s protocol (Promega, Fitchburg, WI, USA). All lab procedures were performed at Mississippi State University, Starkville, MS, USA. We amplified mitochondrial NADH dehydrogenase subunit 4 (ND4) with primer pairs and PCR protocols following protocols described in previous studies [34,35], and the Arizona State University Core Facilities conducted Sanger sequencing on an ABI 3730XL capillary sequencer. Chromatogram files were edited and aligned (SEQUENCHER v5.3; Gene Codes Corp., Ann Arbor, MI, USA), and geographic origin was determined through comparison against georeferenced genetic data using the BLAST algorithm on GenBank [36].

Molecular markers developed for *I. delicatissima* were used to generate genotypes for the two putative Saban Black iguanas [37]. For standardization purposes, eight *Iguana* sp. samples from the IguanaBase dataset analyzed at the GenIndexe Animal Genetics Lab in France, were also genotyped and compared to their original GenIndexe scores [38]. A total of 17 microsatellite markers were evaluated (based on van den Burg et al. [39]), and polymerase chain reaction (PCR) protocols specified by Schuelke [40] were used to amplify DNA segments at the specific marker sites. A 1% agarose gel was used to verify successful PCR amplification. Fragment analysis was conducted by the Cornell Institute of Biotechnology, where microsatellites were sized by an Applied BioSystems 3730XL instrument with the LIZ500 size standard. Genotypes were visualized using PeakScanner™ v 1.0 (Applied Biosystems, Waltham, MA, USA). Allele size ranges were cross examined with the IguanaBase database to ensure scored alleles occurred within the size range of the species [41]. For microsatellite loci, we determined the geographic origin of the pet-trade iguanas through cluster assignment using the predict.dapc() function (*adegenet* package [42]) within the R environment v. 2202.7.1 [43]. Non-hybrid native iguanas in IguanaBase were used as a reference (for georeferenced locations and taxonomy, see Figure S1).

## 3. Results

### 3.1. ND4 Sequence

Mitochondrial ND4 sequences (555 bp in size; GenBank accession OP572228) matched that of the CAR2 haplotype (100%; GenBank accession HM352505). This haplotype is only known to be present in animals native to Saba, Montserrat, and St. Croix and St. Thomas [15,29,30].

### 3.2. Microsatellites

**Figure 1:**
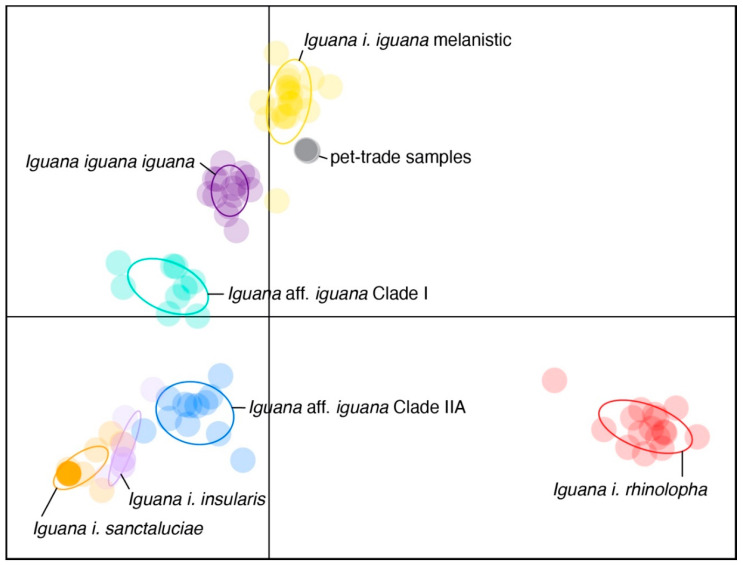
Discriminant principal components scatter plot generated from 17 microsatellite loci representing color-coded individuals from the IguanaBase reference database [41] and two pet-trade individuals (grey). Colored circles indicate group inertia ellipses.

Microsatellite genotypes were determined for 15 of the initial 17 loci (PCR amplifications for L20 and L24 were inconsistent). A subset of animals previously genotyped by GenIndexe were used to ensure consistency in genotyping conducted at MSU. Discriminant analysis of principal components indicated that the two melanic individuals from the pet trade both have multi-locus genotypes consistent with genotypes of animals native to Saba and Montserrat (Figure 1). For markers with no missing data, the pet-trade animals and Saba/Montserrat IguanaBase samples were all fixed for the same alleles (L3, L8, L13, L16, and L17). However, for L23, allele size 308 is novel compared to previously analyzed samples from Saba [41], although documented during a recent population assessment of the Saban iguana population [18]. Additionally, allele 194 for L25 has been previously identified within the native Montserrat population [18].

## 4. Discussion

Despite the extensive trade in *I. iguana*, only a handful of captive pet-trade samples have been analyzed, whereas a larger number of non-native, non-captive iguana samples believed to be released former pet-traded animals, have been used in previous studies [29,30]. Our results strongly suggest the presence of illegal trade in *I. iguana* through fraudulently acquired CITES permits in the absence of any permits from both islands in the CITES database. This forensic application demonstrates the effectiveness of present molecular tools to monitor illegal trade in iguanids (e.g., *Conolophus* [44] and *Ctenosaura* [45]) and highlights the need to apply forensic genetics to the *I. iguana* trade.

Previous studies have highlighted that the trade of melanistic iguanas is likely facilitated through St. Maarten and Barbados to at least seven countries, with St. Maarten being heavily referenced during interviews with several traders [27,28]. Whereas our results suggest that the probable origin of our pet-trade samples is Saba or Montserrat, the mitochondrial haplotypes generated during this study are also present on St. Croix and St. Thomas [30], iguana populations included within the proposed *Iguana melanoderma* [15]. However, all interviewed importing traders and online advertising propose Saba as the native origin of traded melanistic iguanas, with exports from St. Maarten and Barbados. One trader personally traveled to St. Maarten to handle export and import into Europe, further corroborating St. Maarten as a primary facilitator. Further supporting evidence that the traded iguanas do not originate from Montserrat, St. Croix, or St. Thomas but from Saba is suggested by their strong melanistic appearance. Across these islands, all native iguanas are (partially) melanistic, with at least a distinct melanistic patch between the tympanum and eye [15,18,22]. However, body melanism differs considerably between islands, and phenotypes with extensive body melanism equal to that of the traded iguanas appear to only be present on Saba [15,18,22]. The CITES Trade Database for 2020 indicates that no iguanas were exported from Barbados that year, whereas 390 iguanas, of which 320 were captive bred in origin, were exported from St. Maarten to the USA, Japan, Germany, and the Republic of Korea [46]. Data for 2021 are not yet available.

Forensic genetic evidence of illegal trade in iguanas stresses the need for improved enforcement and more robust assessment of CITES applications. Responding to an absence of previous recommendations (e.g., identification guides and regional genetic facilities), a temporary halt on live *Iguana* trade was proposed [47]. If prior management suggestions were implemented, primarily the establishment of within-region genetic infrastructure, trade within the *Iguana* genus could be resumed with mitigated risk to endemic populations targeted by the illegal pet trade. Within-region genetic facilities could be used to assess the geographic origin of iguanas prior to export with low laboratory and technical requirements, including rapid assessment through nanopore technology or fluorescence workflow [48,49].

## 5. Conclusions

Genetic tools should be prioritized over purely morphological methodologies due to the high likelihood of accurate identification and existing native datasets [41,50,51]. Although diagnostic morphological characters distinguishing native *Iguana* populations and species are being studied (e.g., [52]), these efforts should be intensified and extended to include more data on hatchlings and admixed pets (see van den Burg et al. [18] for native vs. non-native populations). This is particularly pertinent considering that exported iguanas are commonly juveniles, which are typically harder to establish geographic origin when compared to adults, especially for untrained assessors. By implementing forensic genetic practices, traders could be discouraged from taking advantage of current CITES regulations, thus restricting the subsequent illegal trade of melanistic iguanas.

## Data Availability

The generated data presented in this study are available within the text and are openly available in FigShare at https://doi.org/10.6084/m9.figshare.13584923.v2 (accessed 1 October 2022) [41].

## Supplementary Materials

The following supporting information can be downloaded at: https://www.mdpi.com/article/10.3390/ani12192660/s1, Figure S1: Map highlighting sample localities and current taxonomic status within Iguana: Inset shows the Lesser Antilles. Codes correspond to sampling locations as mentioned in IguanaBase v2 [41].
