# Supplementary figures and images for: Forensic Genetic Analyses of Melanistic Iguanas Highlight the Need to Monitor the Iguanid Trade
Source: Animals (Basel). 2022 Oct 3;12(19):2660. doi: 10.3390/ani12192660 (PMC9559298; doi:10.3390/ani12192660)

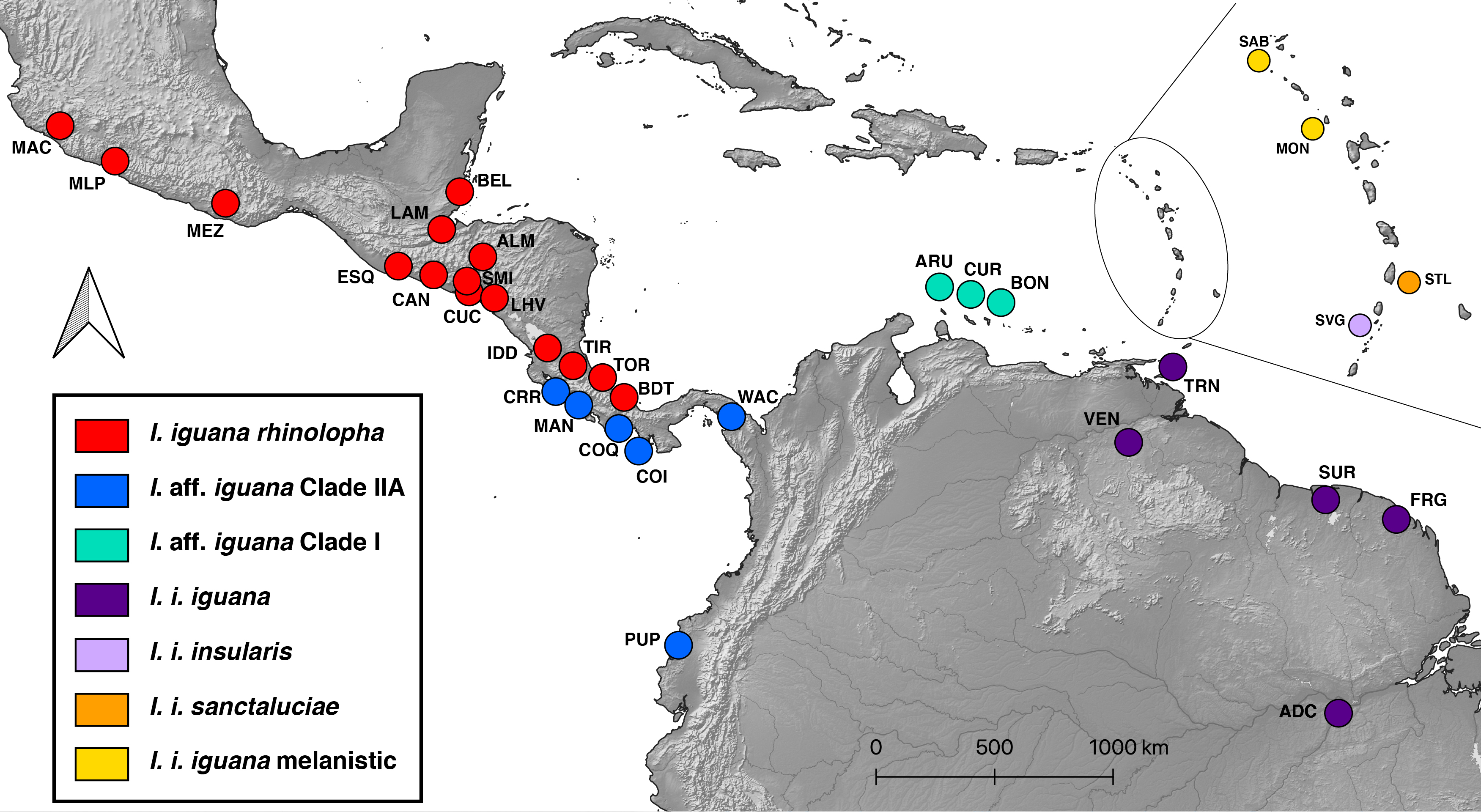

Supplement: Supplementary file 1 [file animals-12-02660-s001.zip › animals-1899063-Supplementary Figure S1.jpg]
